# Reactive Oxygen Species and Male Fertility

**DOI:** 10.3390/antiox9040287

**Published:** 2020-03-29

**Authors:** Cristian O’Flaherty

**Affiliations:** 1 Department of Surgery (Urology Division), McGill University, Montréal, QC H4A 3J1, Canada; 2 Department of Pharmacology and Therapeutics, McGill University, Montréal, QC H3G 1Y6, Canada; 3 The Research Institute, McGill University Health Centre, Montréal, QC H4A 3J1, Canada

Human infertility affects ~15% of couples worldwide, and it is now recognized that in half of these cases, the causes of infertility can be traced to men [1,2]. The spermatozoon is a terminal cell with the unique goal of delivering the paternal genome into the oocyte. This essential task for any species survival can be threatened by environmental pollutants, chemicals, drugs, smoke, toxins, radiation, diseases, and lifestyles. Oxidative stress is a common feature of the mechanism of action of these factors and conditions that negatively impact male fertility [3,4,5,6,7]. Indeed, the reactive oxygen species (ROS)-mediated damage to spermatozoa is a significant contributing factor to infertility in 30-80% of infertile men [8,9,10,11,12].

Spermatozoa are terminally differentiated cells produced in the testes during the hormone-regulated process of spermatogenesis. Two somatic cell types are critical to this process: Sertoli cells protect and support the germ cell development, whereas interstitial Leydig cells produce necessary intratesticular steroids (e.g., testosterone) [13,14]. After their release from the testis, spermatozoa complete their maturation during the transit through the epididymis. There, they acquire the potential for motility and fertility through extensive morphological and biochemical modifications [15]. After ejaculation, spermatozoa must undergo the complex and timely process of capacitation that involves ionic, metabolic, and membrane changes, including the production of ROS at low concentrations [16,17]. Capacitation allows spermatozoa to bind to the zona pellucida that surrounds the oocyte and induce the acrosome reaction [18], an exocytotic event by which proteolytic enzymes (e.g., acrosin and hyaluronidase) are released. Thus, the spermatozoon penetrates the zona pellucida and reaches and fuses with the oocyte. Failure to undergo sperm capacitation and/or acrosome reaction is associated with infertility [19,20].

A key feature of sperm capacitation is the production of ROS at very low and controlled levels by the spermatozoon. This essential phenomenon for the acquisition of the fertility ability was first reported in humans [21], bovine [22], and equine [23] spermatozoa, and then confirmed by others (see [24] for more information). ROS play the role of second messengers and act in most of the known signal transduction pathways involved in this complex phenomenon [25,26,27]. Sperm capacitation is a redox-regulated process. Peroxiredoxins (PRDXs) play a crucial role in maintaining low levels of intracellular ROS to allow the achievement of fertilizing ability by the spermatozoon [28,29].

Oxidative stress can promote detrimental changes during spermatogenesis, epididymal maturation, and sperm capacitation that can lead to infertility [30,31,32,33,34,35]. Lipid peroxidation of the sperm plasma membrane is one of the first described oxidative damage associated with low sperm quality and infertility [36,37]. 4-hydroxynonenal, a subproduct of lipid peroxidation, forms adducts with proteins and DNA, impairing sperm mitochondrial function and promoting mutations in the sperm genome [38]. PRDX6, a unique antioxidant with peroxidase and calcium-independent phospholipase A_2_, is a key element in the antioxidant defence to protect sperm membranes and DNA from oxidative damage [39,40]. Oxidative damage to the paternal genome has been reported in humans and animals and associated with fertility failure [41,42,43,44]. Redox-dependent protein modifications are related to low sperm quality and infertility [45,46,47]. The reproductive system is equipped with antioxidant enzymes to avoid the adverse effects of high levels of ROS during the production and maturation of spermatozoa. Different studies have addressed the consequences of the absence of antioxidant enzymes on male reproduction (for details see [48]), and from knockout mouse models, we learn the critical need of superoxide dismutase, glutathione peroxidases, thioredoxins, and PRDXs to produce a healthy and fertile spermatozoa in young adult and aging males [32,41,49,50,51,52].

The decline of fertility and the increase of abnormalities in the semen of men that is observed over more than two decades is worrisome. However, we still do not have sufficient information to understand why this is happening [53,54,55]. The exposure environmental toxicants could partly explain this phenomenon, but there is still a significant amount of uncertainty regarding the possible causes of the decrease in sperm quality over the years [53,55]. It is an established trend that men are delaying fatherhood due to professional reasons, and scientific data from studies in animals and humans revealed that sperm quality worsens as men age. There is increasing evidence that children fathered by 50-year-old-or older men are prone to manifest a variety of disorders that can be linked to significant mutations of the paternal genome [56].

This Special Issue on ROS and male fertility is composed of four original contributions that provide new data on the role of SOD [57] and PRDXs [58] as essential antioxidants in the epididymis, and PRDXs as novel players in the protection of gonocytes against oxidative stress [59] and the participation of glutathione-S transferase omega 2 in the regulation of sperm function during capacitation [60]. The Special Issue is completed with six review articles that provide an update on the molecular changes induced by oxidative stress that impairs human sperm motility [61], the importance of ROS in determining the functionality of spermatozoa and situations in which oxidative stress occurs and impacts on male fertility [24], the oxidation of the sperm nucleus and the impact of oxidative stress on the paternal genome [62], the importance of using appropriate tools to study the role of ROS in sperm and infertility [63], and an exhaustive evaluation of ROS production and its relevance in male fertility and antioxidant therapy [64]. The characterization of the redox regulation and effects of oxidative stress in equine spermatozoa is also included in this Special Issue [65].

Many groundbreaking studies have contributed to our understanding of the field of ROS in male fertility. It is undeniable that these reactive molecules play an essential role in both physiological and pathological mechanisms of the male reproductive system. Antioxidant therapy is a common clinical strategy to improve sperm quality and function in infertile men [66,67]. However, there are controversial results regarding the efficacy of these treatments. Some controlled trials suggested that such treatments are beneficial to achieve live births [66], whereas others did not show any benefit [68]. This discrepancy is likely based on the lack of tools in clinics to establish whether oxidative stress is responsible for the infertility of a given patient. Thus, more fundamental and clinical research is needed to comprehend how ROS modulate male fertility to design better diagnostic tools and therapeutic strategies to fight against male infertility.
